# Mitochondrial‐derived peptides MOTS‐c and humanin attenuate dexamethasone‐induced atrophy in human skeletal muscle cells

**DOI:** 10.14814/phy2.70791

**Published:** 2026-02-24

**Authors:** Rabie Elhusseiny, Mohammed Ihsan, Théo Bellefroid, Abdulaziz Farooq, Sébastien Racinais, Louise Deldicque

**Affiliations:** ^1^ Institute of Neuroscience UCLouvain Louvain‐la‐Neuve Belgium; ^2^ Physical Education Department, College of Education United Arab Emirates Université Al Ain UAE; ^3^ Aspetar Orthopaedic and Sports Medicine Hospital Research and Scientific Support Department Doha Qatar; ^4^ DMEM Univ Montpellier, INRAE Montpellier France; ^5^ Environmental Stress Unit CREPS Montpellier Font‐Romeu Montpellier France

**Keywords:** atrogenes, fusion index, glucocorticoid, mitokines, myotubes

## Abstract

Glucocorticoids, such as dexamethasone (DEXA), are effective therapeutics but cause severe muscle wasting. Mitochondrial‐derived peptides (MDPs) are promising countermeasures, but their effectiveness is largely unexplored. We tested the hypothesis that the MDP S14G‐humanin (HNG) and the mitochondrial open reading frame of the 12S rRNA‐c (MOTS‐c) mitigate DEXA‐induced atrophy in human skeletal myotubes. Fully differentiated primary human myotubes were exposed to 10 μM DEXA ±10 μM HNG or 10 μM MOTS‐c. DEXA decreased myotube size (area, *p* < 0.001) and differentiation (Fusion Index, *p* = 0.05). Additionally, DEXA increased both muscle ring finger protein 1 (MURF1, *p* < 0.001) and muscle atrophy F‐box (MAFbx, *p* = 0.01) as well as peroxisome proliferator‐activated receptor‐gamma coactivator‐1 alpha (PGC1α, *p* < 0.001). MOTS‐c co‐treatment with DEXA completely preserved myotube area (*p* < 0.001) and fusion index (*p* = 0.02), increased Akt phosphorylation (*p* = 0.0015) and blunted both MURF1 upregulation (*p* = 0.03) and STAT3 activation (*p* = 0.005) compared to DEXA alone. HNG co‐treatment with DEXA preserved myotube area (*p* < 0.001), blunted DEXA‐induced STAT3 activation (*p* = 0.027), but had no effect on fusion index or E3 ligase mRNA levels. Those findings suggest that MOTS‐c could be an effective inhibitor of glucocorticoid‐induced atrophy in human muscle, not only through selective inhibition of MURF1 but also by enhancing Akt signaling and suppressing STAT3 activation.

## 1 INTRODUCTION

Muscle atrophy is a serious condition characterized by decrease in muscle mass, strength, and function. It can result from a variety of causes, such as disuse, denervation, aging, diseases, and as a side effect of some therapeutic treatments. A notable example is glucocorticoid‐induced muscle atrophy, which arises following exposure to synthetic glucocorticoids like dexamethasone (DEXA). Although these glucocorticoids are broadly prescribed for their potent anti‐inflammatory and immunosuppressive actions, their long‐term or high‐dose administration causes severe side effects, among which includes muscle atrophy. For this reason, DEXA is frequently used as a reliable model of muscle atrophy in both animal and in vitro studies (Alonso‐Puyo et al., 2024; Zhang et al., 2024). The catabolic action of DEXA is mainly mediated through inhibiting protein synthesis and stimulating muscle proteolysis. This occurs via a glucocorticoid receptor‐mediated process where DEXA induces Krüppel‐like factor 15 (KLF15), which upregulates the key ubiquitin E3 ligases, muscle atrophy F‐box (MAFbx) and muscle ring finger protein 1 (MURF1), promoting protein degradation (Ulla et al., 2021).

Recently, a new class of bioactive peptides, known as mitochondrial‐derived peptides (MDPs), encoded by the mitochondrial genome, has emerged as an exciting area of investigation. These short peptides include humanin (HN), small humanin‐like peptides (SHLPs), and the mitochondrial open reading frame of the 12S rRNA‐c (MOTS‐c), all encoded by short open reading frames (ORFs) found in mitochondrial DNA (Gao et al., 2023). MDPs play critical roles in the processes of aging, metabolic disease, and cardiovascular function, with circulating levels of MDPs known to decrease in conditions of type 2 diabetes and with age (Gong et al., 2022).

HN, the first‐described and most‐characterized MDP, is a 24‐amino‐acid polypeptide coded by the MT‐RNR2 gene in the 16S rRNA mtDNA region. Originally identified for its neuroprotective action in the surviving neurons of Alzheimer's patients, HN is found in a variety of tissues, including brain and skeletal muscle, and confers general cytoprotective actions (Niikura et al., 2003). Intracellularly, HN has been shown to prevent apoptosis by binding pro‐apoptotic proteins such as BAX and tBid. Extracellularly, HN is secreted and binds two primary classes of cell surface receptors to initiate pro‐survival signaling, through among others the JAK2/STAT3 signaling pathway (Zhu et al., 2022). Synthetic analogs of HN with increased potency, for example, S14G‐HN (HNG), have been created to augment its therapeutic potential (Zhu et al., 2022).

MOTS‐c is another MDP, a 16‐amino‐acid peptide coded by the 12S rRNA region of the mitochondrial genome. It acts as an important regulator of cellular homeostasis, especially under metabolic stress. To exert its cytoprotective actions, MOTS‐c relocates from the mitochondria to the nucleus, where it directly controls the transcription of nuclear genes to achieve metabolic balance (Zheng et al., 2023). It is expressed in muscle tissue and plasma, where the levels of circulating MOTS‐c decline with age (Gao et al., 2023).

Despite the established protective effects of these peptides, their precise role in opposing glucocorticoid‐induced muscle atrophy is largely unexamined. Prior studies have shown that HNG is able to protect chondrocytes against DEXA‐induced apoptosis, as well as blunt the negative effects of glucocorticoids on bone growth (Celvin et al., 2020; Zaman et al., 2019). No studies have examined the impact of HN or its analogs on muscle cells in the context of glucocorticoid‐induced atrophy. Likewise, although MOTS‐c has proven benefits for muscle metabolism, its role as a direct therapeutic agent against muscle wasting is not well defined. Recent evidence has shown that MOTS‐c can attenuate disuse‐induced muscle atrophy in rodent models, such as those involving hindlimb unloading or denervation (Kumagai et al., 2021; Kumagai, Kim, Miller, Natsume, et al., 2024; Kumagai, Kim, Miller, Zempo, et al., 2024). This anti‐atrophic potential, demonstrated in vivo, suggests MOTS‐c is a promising therapeutic candidate for various muscle wasting conditions. Despite these observations, its use in the treatment of disease is still uncommon, and its precise mechanism for opposing glucocorticoid‐induced muscle atrophy in human cells remains unexplored. A substantial amount of research on the molecular mechanisms of muscle wasting has been conducted using animal models, particularly rodents (Mueller et al., 2016). However, the poor translation of findings from these preclinical studies to effective human therapies highlights a critical disconnect. This translational gap underscores the need to shift focus toward models that more closely recapitulate human physiology.

Thus, the aim of this study was to explore the possibility of the mitochondrial‐derived peptides HNG and MOTS‐c to mitigate DEXA‐induced atrophy in an in vitro model of primary human skeletal muscle myotubes. We hypothesized that HNG and MOTS‐c would mitigate the catabolic effects of DEXA. To test this, we evaluated myotube morphology and investigated the underlying molecular mechanisms by examining changes in the expression of key muscle atrophy markers, mitochondrial function, and important anabolic and catabolic signaling pathways.

## 2 MATERIALS AND METHODS

### 2.1 Human skeletal muscle cell culture and differentiation

Human skeletal muscle cells, isolated from the quadriceps of 2 donors from the young sedentary group as detailed in Balan et al. (Balan et al., 2020) and cryopreserved at passage 3 in the laboratory, were used for all experiments. No difference in the morphological or molecular responses was observed between the 2 donors, which were used interchangeably in the different experiments. Cryovials were thawed quickly in a 37°C water bath, and cells were seeded on 100‐mm dishes in proliferation medium. The latter was composed of Dulbecco's Modified Eagle Medium (DMEM; Biowest, Nuaillé, France) with 20% (v/v) fetal bovine serum (FBS; Sigma‐Aldrich, Taufkirchen, Germany), 0.5% (v/v) Ultroser G (15950‐017, Pall Life Sciences, Cergy, France), and 1% (v/v) penicillin–streptomycin (VWR, Radnor, PA, USA). Cultures were kept in a humidified incubator at 37°C with 5% CO_2_. The medium was changed within 24 h after thawing to eliminate residual DMSO and then refreshed every 48 h. At 50% confluence, cells were passaged by standard trypsinization procedures. For myogenic differentiation, myoblasts were cultured to 100% confluence and then changed to differentiation medium, which was made up of DMEM supplemented with 2% (v/v) FBS and 1% (v/v) penicillin–streptomycin. Cells were kept in differentiation medium for 4 days to enable the development of mature multinucleated myotubes prior to treatments.

### 2.2 Induction of myotube atrophy and peptide treatment

Differentiated myotubes were allocated to one of the six treatment groups: (1) vehicle control, (2) 10 μM HNG (H6161, Sigma‐Aldrich, Taufkirchen, Germany), (3) 10 μM MOTS‐c (AS‐65587, Eurogentec, Seraing, Belgium), (4) 10 μM DEXA (D4902, Sigma‐Aldrich, Taufkirchen, Germany), (5) 10 μM DEXA + 10 μM HNG, or (6) 10 μM DEXA + 10 μM MOTS‐c. The concentration and duration for all agents were optimized in preliminary experiments. DEXA doses ranging from 0.1 μM to 100 μM were tested over 6, 24, and 48 h to reliably induce a significant atrophic phenotype, with 10 μM DEXA and a 24‐h exposure period selected for the main study. Similarly, HNG and MOTS‐c concentrations (0.1, 1, and 10 μM) were tested based on their modulatory effects on *MURF1*, *MAFbx*, and *myostatin* gene expression, leading to the selection of 10 μM for subsequent peptide treatments. Peptides (HNG or MOTS‐c) were applied for 6 h prior to the addition of DEXA and then co‐incubated for the subsequent 24 h (a total duration of 30 h). This pre‐treatment step was implemented to ensure the MDPs had sufficient time to initiate their cytoprotective effects and allow us to test their capacity to prevent atrophy before the full catabolic stress of the glucocorticoid was imposed. For the groups treated with HNG or MOTS‐c alone (without DEXA), the total MDP treatment duration was also 30 h. The cells were then harvested for biochemical or morphological analyses.

### 2.3 Immunocytochemistry and morphological analysis

After treatment, cells were washed in phosphate‐buffered saline (PBS, pH 7.4) and fixed in 4% (w/v) paraformaldehyde in PBS for 20 min at room temperature. Following fixation, cells were permeabilized using 0.2% (v/v) Triton X‐100 in PBS for 15 min. Non‐specific antibody binding was blocked for 30 min using PBS with 0.5% (w/v) bovine serum albumin (BSA, A9647, Sigma‐Aldrich) and 5% (v/v) goat serum. Myotubes were visualized by incubating cells with a primary desmin antibody (Clone D33, Cat# M0760; Dako/Agilent, Santa Clara, CA, USA; 1:100 dilution) for 1 h at room temperature. Cells were washed before incubation with a goat anti‐mouse IgG secondary antibody conjugated to Alexa Fluor 488 (Cat# ab150113; Abcam, Cambridge, UK; 1:500 dilution) for 1 h in the dark. Nuclei were counterstained using VECTASHIELD® Antifade Mounting Medium with DAPI (Cat# H‐1200; Vector Laboratories, Burlingame, CA, USA).

Images were taken on a fluorescence microscope (Zeiss Axio A1, Oberkochen, Germany) equipped with an AxioCam ICm1 camera (10× magnification) and ZEN lite 2011 (Zeiss) software. Myotube morphology was analyzed using the MyoCount software (Murphy et al., 2019). Two parameters were measured: (1) myotube area, as the percentage of the total image area that was stained positive for desmin, and (2) the fusion index, as the percentage of nuclei found within desmin‐positive myotubes (containing ≥3 nuclei) out of the total number of nuclei.

### 2.4 Quantitative real‐time polymerase chain reaction

Total RNA was isolated from myotubes using TRIzol® reagent (Invitrogen, Carlsbad, CA, USA) according to the manufacturer's protocol. RNA concentration and purity were determined by measuring absorbance at 260/280 nm using a NanoDrop™ 2000 spectrophotometer (Thermo Fisher Scientific, Waltham, MA, USA). For each sample, 1 μg of total RNA was reverse transcribed into complementary DNA (cDNA) using the iScript™ cDNA Synthesis Kit (1708891, Bio‐Rad Laboratories, Hercules, CA, USA).

Quantitative real‐time polymerase chain reaction (qRT‐PCR) was conducted on the CFX96 Real‐Time PCR Detection System (Bio‐Rad Laboratories). Reactions were set up in a 10 μL final reaction volume, comprising 5 μL of appropriately diluted cDNA, 4.8 μL SsoAdvanced™ Universal SYBR® Green SuperMix (1725274, Bio‐Rad), and 0.1 μL of each primer (final concentration 100 nM). Primer sequences are listed in Table 1 and were supplied from Integrated DNA Technologies (Leuven, Belgium). Thermal cycling conditions were: 95°C for 3 min, then 40 cycles of 95°C for 15 s and 60°C for 30 s. Relative gene expression was determined by the 2^−ΔΔCT^ method, using beta‐2‐microglobulin (β2M) as the endogenous housekeeping gene.

**TABLE 1:** List of primers used for real time PCR.

	Forward	Reverse
B2M	ATG AGT ATG CCT GCC GTG TGA	GGC ATC TTC AAA CCT CCA TG
MAFbx	GTG GTA CTG AAA GTC CTT GAA	CTC TTT GGA CCA GTG TAC ATA A
MURF1	CTA TCT GCC TGG AGA TGT TTA C	TTT GCA GCC TGG AAG ATG
Myostatin	TTG ACA TGA ACC CAG GCA CTG GTA	AAC GGA TTC AGC CCA TCT TCT CCT
PGC1α	CAC TTA CAA GCC AAA CCA ACA ACT	CAA TAG TCT TGT TCT CAA ATG GGG A
TFAM	CTC AGA ACC CAG ATG CAA A	GCC ACT CCG CCC TAT AA

Abbreviations: B2M, beta 2 microglobulin; MAFbx, muscle atrophy F‐box; MURF1, muscle‐specific RING finger protein 1; PGC1α, peroxisome proliferator‐activated receptor gamma coactivator 1 alpha; TFAM, mitochondrial transcription factor A.

### 2.5 Protein immunoblotting

Cells were lysed on ice with 75 μL of lysis buffer (20 mM Tris–HCl pH 7.0, 270 mM sucrose, 5 mM EGTA, 1 mM EDTA, 1 mM sodium orthovanadate, 50 mM β‐glycerophosphate, 5 mM sodium pyrophosphate, 50 mM sodium fluoride, 1 mM dithiothreitol, and 1% Triton‐X 100) supplemented with cOmplete™ Protease Inhibitor Cocktail (4693124001, Roche, Basel, Switzerland). Lysates were scraped, collected, and clarified by centrifugation at 10,000×*g* for 10 min at 4°C. The supernatant was collected, and protein concentration was determined using the DC™ Protein Assay (Bio‐Rad Laboratories, Hercules, CA, USA) against a BSA standard curve.

Equal amounts of protein were loaded and resolved by SDS‐PAGE on 10%–15% polyacrylamide gels and transferred onto polyvinylidene difluoride (PVDF) membranes (1620177, Bio‐Rad). Membranes were stained with Ponceau S (P3504, Sigma‐Aldrich) to ensure transfer efficiency prior to blocking for 1 h at room temperature in Tris‐buffered saline with 0.1% Tween‐20 (TBST) and 5% (w/v) non‐fat dry milk. Membranes were incubated overnight at 4°C with primary antibodies diluted in TBST containing 0.1% (w/v) BSA or non‐fat dry milk. The following primary antibodies were obtained from Cell Signaling Technology (Denvers, MA, USA): Akt (#9272), p‐Akt (Ser473) (#4060), NF‐κB p65 (#8242), p‐NF‐κB p65 (Ser536) (#3033), STAT3 (#12640), p‐STAT3 (Tyr705) (#9131), caspase‐3 (#14220), and eEF2 (#2332) and from Abcam (Cambridge, UK): OXPHOS Rodent WB Antibody Cocktail (#ab110413).

Following washing, membranes were incubated with HRP‐conjugated goat anti‐rabbit (A6154) or anti‐mouse (A9044) secondary antibodies (Sigma‐Aldrich, 1:10,000 dilution) for 1 h at room temperature. Protein bands were detected using Immobilon Forte Western HRP Substrate (WBLUF0100, Millipore, Burlington, MA, USA) and were imaged on a GeneSnap system (Syngene, UK). Band intensities were measured using GelAnalyzer software (v. 19.1). Target protein expression was normalized to eukaryotic elongation factor 2 (eEF2) as a reference protein or to the respective total form when a phosphorylated form was analyzed.

### 2.6 Statistical analysis

Data are presented as mean ± standard deviation (SD). Each dot represents one experiment, which was replicated in 3 independent cultures (*N* = 3). Statistical analysis was carried out with a one‐way analysis of variance (ANOVA) followed by Fisher's least significant difference (LSD) post‐hoc test for multiple group comparisons among the six treatment groups. The magnitude of the overall effect for the ANOVA (effect size) was determined using eta‐squared (*η* ^2^). Values ≥0.14 were considered a large effect. A *p*‐value <0.05 was deemed statistically significant. All statistical analyses were conducted with SPSS Statistics for Windows (Version 28.0; IBM Corp., Armonk, NY, USA).

## 3 RESULTS

### 3.1 MOTS‐c and HNG rescue dexamethasone‐induced myotube atrophy

**FIGURE 1:**
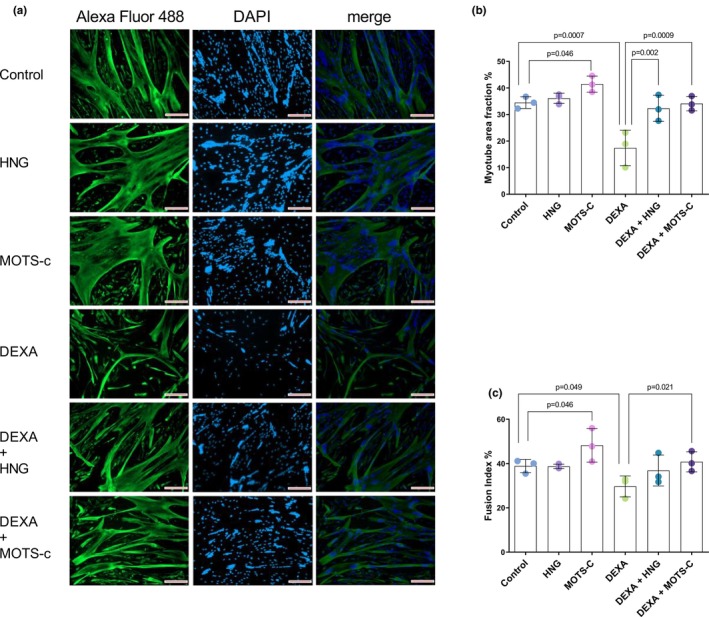
MOTS‐c and HNG mitigate dexamethasone‐induced myotube atrophy. Primary human skeletal muscle myotubes were treated with vehicle (Control), 10 μM S14G‐humanin (HNG), 10 μM mitochondrial open reading frame of the 12S rRNA‐c (MOTS‐c), 10 μM dexamethasone (DEXA), or co‐treated with DEXA and either HNG or MOTS‐c. (a) Representative immunofluorescence images of myotubes stained for desmin (green, Alexa Fluor 488) to visualize myotube structure, with nuclei counterstained with DAPI (blue), a red scale bar represents 100 μm. (b) Quantification of myotube area fraction, calculated as the percentage of the total image area positive for desmin staining. (c) Quantification of the fusion index, calculated as the percentage of nuclei within myotubes (containing ≥3 nuclei) relative to the total number of nuclei.

To examine the effect of MOTS‐c and HNG on skeletal muscle phenotype, we initially assessed myotube morphology in response to treatment with DEXA (Figure 1a). One‐way ANOVA and size effect analysis revealed a large overall effect of the treatments on myotube area fraction (*F*(5, 12) = 12.51, *p* < 0.001; *η* ^2^ = 0.839). Post‐hoc analysis showed that DEXA treatment caused a decrease in myotube size, as quantified by the myotube area fraction, relative to vehicle‐treated control cells (17.4% ± 6.7% vs. 34.5% ± 2.2%, *p* < 0.001; Figure 1b). MOTS‐c treatment increased the myotube area fraction above control levels (41.5% ± 3.0% vs. 34.5% ± 2.2%; *p* = 0.046), while it was not the case for HNG (36.1 ± 1.9). Co‐treatment with DEXA and MOTS‐c fully protected against atrophy. The myotube area fraction in the DEXA+MOTS‐c group (34.1% ± 2.7%) was not different from the control group (*p* = 0.993) and was higher than in the DEXA alone group (*p* < 0.001). Similarly, co‐treatment with DEXA and HNG also preserved the myotube area fraction (32.3% ± 4.9%), which was higher than in the DEXA alone group (*p* < 0.001), but was not different from the control group (*p* = 0.981) (Figure 1b).

In agreement with these morphological alterations, an overall effect of treatment was observed on myoblast fusion (*F*(5, 12) = 4.06, *p* = 0.021), with the treatments accounting for a large proportion of the variance (*η* ^2^ = 0.628). DEXA treatment disrupted myoblast fusion, as revealed by a decreased fusion index relative to the control group (29.7% ± 4.8% vs. 38.9% ± 3.0%; *p* = 0.049; Figure 1c). Conversely, MOTS‐c treatment alone stimulated myoblast fusion (48.2% ± 7.6% vs. 38.9% ± 3.0%; *p* = 0.047 vs. control). Co‐treatment with MOTS‐c fully restored the DEXA‐induced decrease in fusion index (40.8% ± 4.5% vs. 29.7% ± 4.8%; *p* = 0.022 vs. DEXA alone), which was higher than in the DEXA alone group (*p* = 0.022), and not different from the control group (*p* = 0.843). Co‐treatment with DEXA and HNG attenuated the decline in fusion index (36.8% ± 7.0%), which tended to be higher than in the DEXA alone group (*p* = 0.111), and was also not different from the control group (*p* = 0.911).

### 3.2 MOTS‐c selectively decreases gene expression of MURF1

**FIGURE 2:**
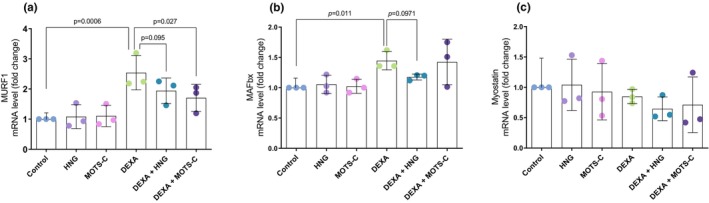
mRNA levels of MURF1 (a), MAFbx (b), and myostatin (c). Primary human skeletal muscle myotubes were treated with vehicle (Control), 10 μM S14G‐humanin (HNG), 10 μM mitochondrial open reading frame of the 12S rRNA‐c (MOTS‐c), 10 μM dexamethasone (DEXA), or co‐treated with DEXA and either HNG or MOTS‐c. Data are presented as fold change relative to the control group and are shown as mean ± SD (*N* = 3). MAFbx, muscle atrophy F‐box; MURF1, muscle‐specific RING finger protein 1.

To investigate the molecular mechanisms underlying the observed atrophy, we measured the expression of major muscle‐specific E3 ubiquitin ligases. One‐way ANOVA showed a large treatment effect on both MURF1 (*F*(5, 12) = 6.82, *p* = 0.003; *η* ^2^ = 0.740) and MAFbx (*F*(5, 12) = 3.61, *p* = 0.032; *η* ^2^ = 0.600) mRNA levels. Post‐hoc tests revealed that DEXA treatment increased the expression of MURF1 by ~2.5‐fold compared to control (2.5 ± 0.6 vs. 1.0 ± 0.0, *p* < 0.001; Figure 2a). MOTS‐c co‐treatment effectively blunted this DEXA‐stimulated upregulation of MURF1 (1.7 ± 0.5 vs. 2.5 ± 0.6; *p* = 0.025 vs. DEXA alone). For HNG co‐treatment, a comparable but non‐significant trend was detected (1.9 ± 0.4 vs. 2.5 ± 0.6; *p* = 0.095 vs. DEXA alone). DEXA also resulted in an upregulation of MAFbx mRNA levels relative to the control group (1.5 ± 0.2 vs. 1.0 ± 0.0; *p* = 0.010; Figure 2b). In contrast to its effect on MURF1, MOTS‐c co‐treatment did not decrease DEXA‐induced upregulation of MAFbx mRNA levels, while HNG co‐treatment induced a trend toward lower MAFbx levels compared to DEXA alone (*p* = 0.097). The observed morphological effects appear to be independent of myostatin, as no differences in its expression were found among any of the treatment groups (*F*(5, 12) = 0.68, *p* = 0.647; *η* ^2^ = 0.221; Figure 2c).

### 3.3 Dexamethasone upregulates PGC‐1α mRNA levels

**FIGURE 3:**
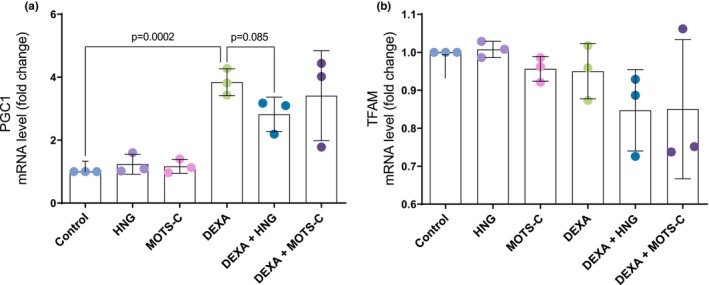
mRNA levels of PGC1α (a) and TFAM (b). Primary human skeletal muscle myotubes were treated with vehicle (Control), 10 μM S14G‐humanin (HNG), 10 μM mitochondrial open reading frame of the 12S rRNA‐c (MOTS‐c), 10 μM dexamethasone (DEXA), or co‐treated with DEXA and either HNG or MOTS‐c. Data are presented as fold change relative to the control group and are shown as mean ± SD (*N* = 3). PGC1α, peroxisome proliferator‐activated receptor‐gamma coactivator‐1 alpha: TFAM, mitochondrial transcription factor A.

**FIGURE 4:**
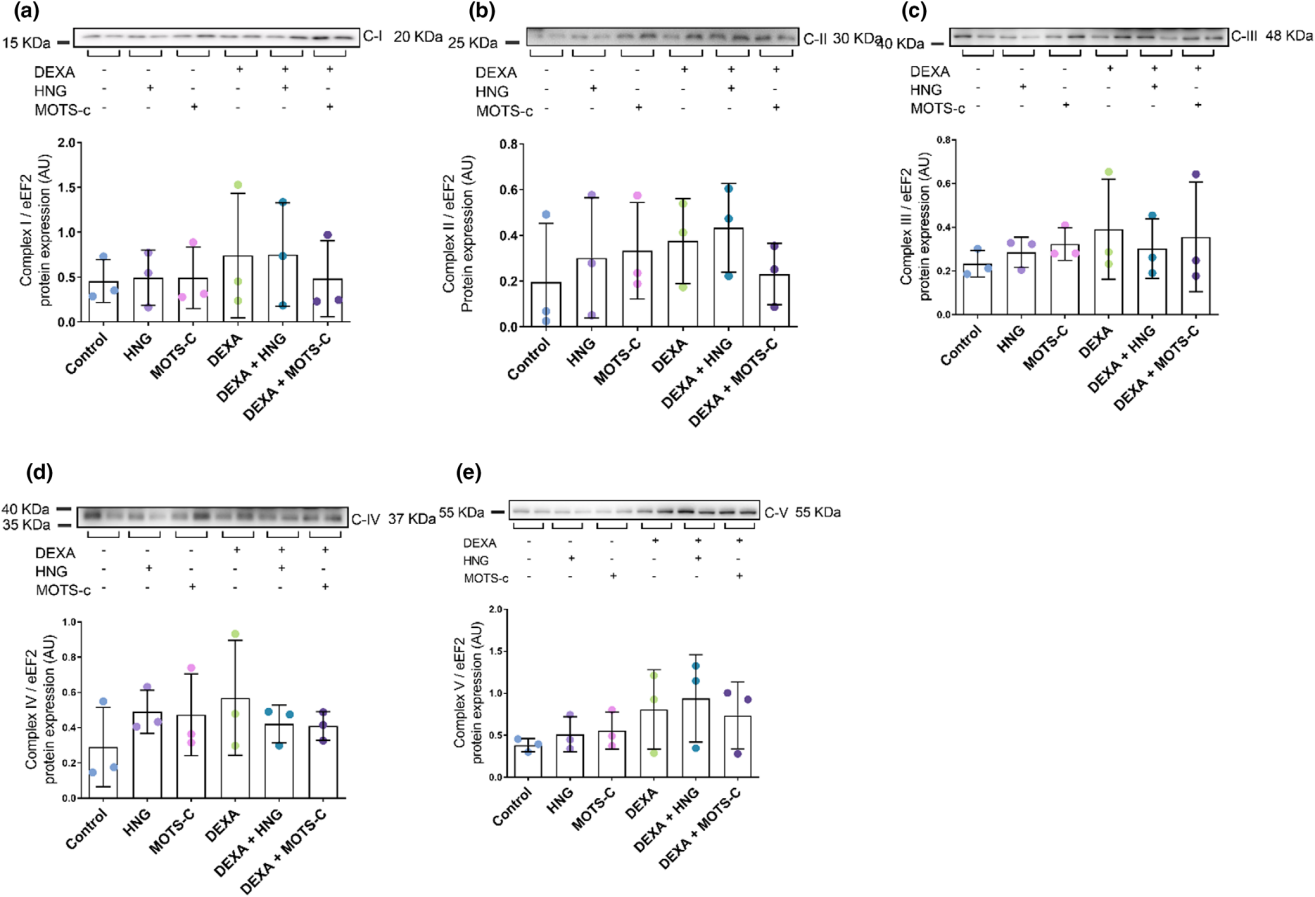
Protein expression of mitochondrial electron transport chain complexes (ETC), Complex I (a), Complex II (b), Complex III (c), Complex IV (d) and Complex V (e), all normalized to eEF2. Primary human skeletal muscle myotubes were treated with vehicle (Control), 10 μM S14G‐humanin (HNG), 10 μM mitochondrial open reading frame of the 12S rRNA‐c (MOTS‐c), 10 μM dexamethasone (DEXA), or co‐treated with DEXA and either HNG or MOTS‐c. Results are presented as mean ± SD (*N* = 3).

We then examined markers of mitochondrial biogenesis. One‐way ANOVA showed a strong overall treatment effect on peroxisome proliferator‐activated receptor‐gamma coactivator‐1 alpha (PGC‐1α) mRNA levels (*F*(5, 12) = 10.73, *p* < 0.001), which accounted for 81.7% of the total variance (*η* ^2^ = 0.817). DEXA treatment caused a robust ~3.8‐fold induction of PGC1α relative to the control group (3.8 ± 0.4 vs. 1.0 ± 0.0, *p* < 0.001; Figure 3a). This induction tended to be partially reversed by co‐treatment with HNG (*p* = 0.085 vs. DEXA alone), though PGC1α mRNA levels remained significantly elevated compared to control (*p* ≤ 0.001 vs. control for both HNG and MOTS‐c). There were no changes in the expression of the downstream target mitochondrial transcription factor A (TFAM) (*F*(5, 12) = 1.74, *p* = 0.200; *η* ^2^ = 0.420; Figure 3b) or in the protein levels of mitochondrial respiratory chain complexes I–V (all *p* > 0.05; Figure 4 a–e).

### 3.4 MOTS‐c and HNG modulate Akt and STAT3 phosphorylation

**FIGURE 5:**
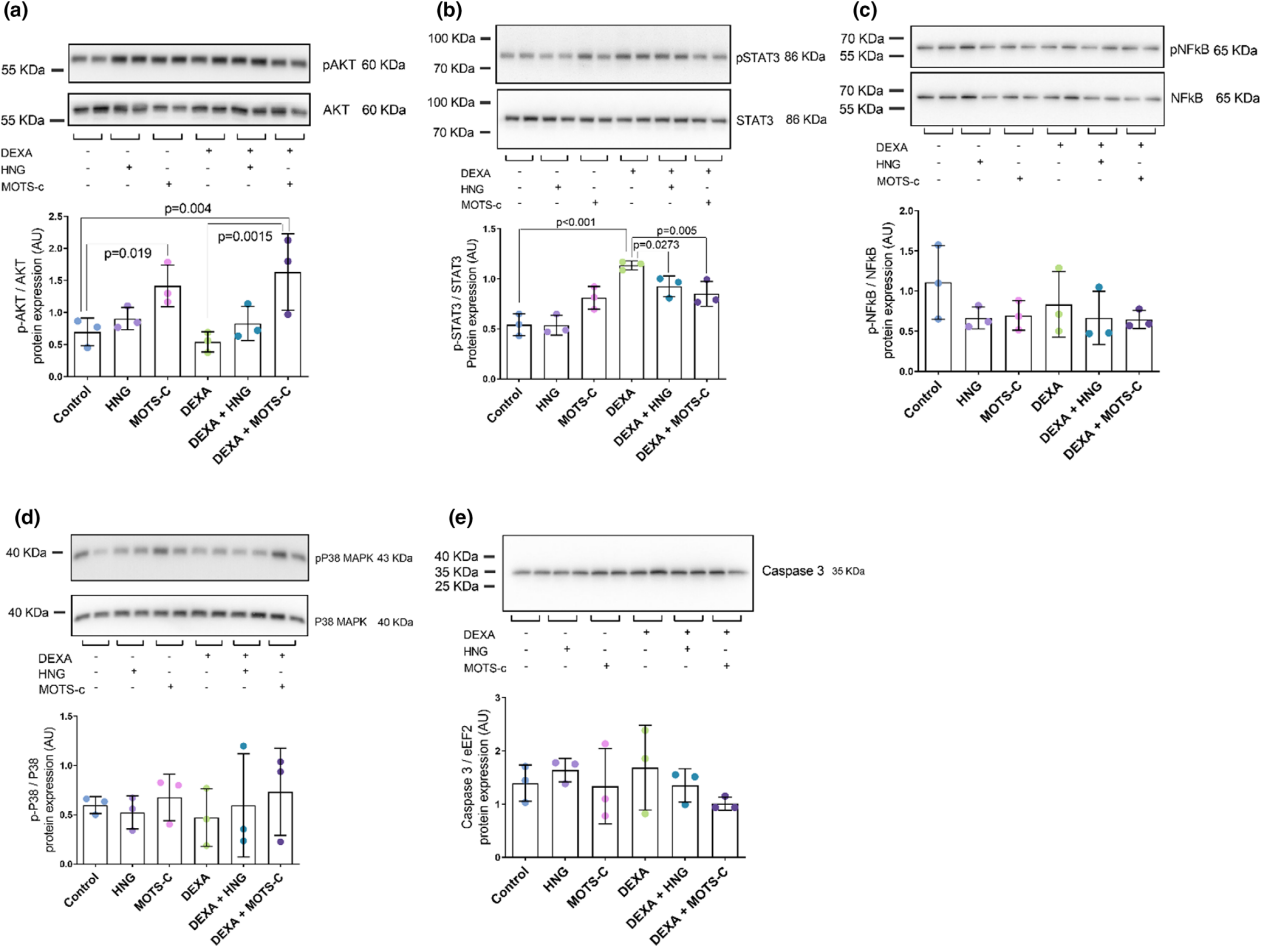
Protein expression of p‐Akt normalized to total Akt (a), p‐STAT3 normalized to total STAT3 (b), p‐NFκB normalized to total NFκB (c), p‐p38 normalized to total p38 (d), and caspase 3 normalized to eEF2 (e). Primary human skeletal muscle myotubes were treated with vehicle (Control), 10 μM S14G‐humanin (HNG), 10 μM mitochondrial open reading frame of the 12S rRNA‐c (MOTS‐c), 10 μM dexamethasone (DEXA), or co‐treated with DEXA and either HNG or MOTS‐c. Results are presented as mean ± SD (*N* = 3).

Analysis of phosphorylation to total form ratios revealed significant treatment effects for Akt (*F*(5, 12) = 5.16, *p* = 0.009, *η* ^2^ = 0.68, Figure 5a) and STAT3 (*F*(5, 12) = 15.36, *p* < 0.001, *η* ^2^ = 0.86, Figure 5b). While DEXA alone did not decrease the p‐Akt/Akt ratio compared to control, co‐treatment with DEXA + MOTS‐c induced a higher ratio compared to both the control group (*p* = 0.004) and the DEXA alone group (*p* = 0.001). DEXA increased the p‐STAT3/STAT3 ratio compared to control (*p* < 0.0001). Both HNG (*p* = 0.027) and MOTS‐c (*p* = 0.005) attenuated this DEXA‐induced STAT3 activation. No changes were observed in the phosphorylation to total form ratios for NFκB (*p* = 0.433) (Figure 5c) or p38 (*p* = 0.929) (Figure 5d) nor in the expression of the apoptotic marker caspase 3 (Figure 5e).

## 4 DISCUSSION

In this study, we investigated the potential of the mitochondrial‐derived peptides HNG and MOTS‐c to counteract glucocorticoid‐induced muscle atrophy in a primary human skeletal muscle cell model. Our findings show that although both peptides can improve the DEXA‐induced atrophic effects, they seem to act via different mechanisms, with MOTS‐c having broader protective action.

The first step of our investigation was to confirm a robust atrophic phenotype in our in vitro model of human skeletal muscle cells following glucocorticoid treatment. Treatment with 10 μM DEXA caused a marked atrophic phenotype, which was characterized by a significant reduction in both myotube area fraction and fusion index. These morphological changes are consistent with a vast literature that reports similar effects in cultured human myotubes (Langendorf et al., 2020; Park et al., 2024), animal models such as gilts, rats, and *Caenorhabditis elegans* (Bae et al., 2025; Otrocka‐Domagała et al., 2019), and rodent cell lines such as the rat‐derived L6 cell line (Menconi et al., 2008) and mouse‐derived cell line C2C12 (Akpulat, 2023; Chang & Kong, 2020). Notably, our finding that DEXA significantly reduces the fusion index, which replicates findings in C2C12 (Bae et al., 2025; Men et al., 2024) and primary rat myotubes (Syverud et al., 2016), constitutes the first report of such an effect in a primary human skeletal muscle cell system confirming a cross‐species conservation of this catabolic mechanism.

At the mechanistic level, this DEXA‐induced atrophy was accompanied by an upregulation of key E3 ligases, with a 2.5‐fold increase in MURF1 and a 1.5‐fold increase in MAFbx mRNA levels. This finding is in concordance with a large portfolio of previous reports in C2C12 myotubes (Hah et al., 2023; Kwag et al., 2025), L6 myotubes (Castillero et al., 2013), primary human myoblasts (Langendorf et al., 2020), and animal models (Nakashima et al., 2016; Sun et al., 2014). Our finding confirms the critical role of the ubiquitin‐proteasome pathway as a primary, conserved mechanism underlying glucocorticoid‐induced muscle wasting across different species. However, the use of a human primary cell model in our research revealed critical, species‐specific differences from the traditional rodent models for glucocorticoid activity, such as PGC‐1α.

A key finding is the significant upregulation of PGC‐1α mRNA levels following DEXA treatment in human myotubes. This is particularly noteworthy as it contrasts with the consistent downregulation of PGC‐1α observed in rodents (Mohammed et al., 2019; Qin et al., 2010; Rizk et al., 2023). This PGC1α suppression has long been recognized as a fundamental mechanism underlying glucocorticoid‐induced atrophy in rodent models. Our observation of PGC1α upregulation in a human cell context indicates a fundamental, species‐specific difference in how skeletal muscle responds to glucocorticoids. This finding has important implications for translational research, particularly since rodent models are frequently employed to inform our understanding of muscle atrophy and to predict human responses. This finding, while unexpected, is not without precedent in other tissues, where DEXA has been reported to increase PGC1α levels in rat aortic smooth muscle cells, rat liver, and fetal mouse heart tissue (Rog‐Zielinska et al., 2015; Tiao et al., 2011; Xu et al., 2011).

Looking at the activation of several pathways known to directly or indirectly regulate protein metabolism, our data revealed that MOTS‐c and HNG modulated some of them. More specifically, MOTS‐c appeared to exert a potent anabolic stimulus by upregulating Akt phosphorylation beyond control levels even in the presence of DEXA. This provides a mechanistic explanation for the increased myotube area observed with MOTS‐c treatment. Furthermore, our data showed that DEXA induced muscle stress through the STAT3 pathway in human muscle cells. Since STAT3 activation promotes protein degradation and muscle atrophy (Sala & Sacco, 2016), the ability of both MOTS‐c and HNG to blunt STAT3 activation suggests that their protective effects are partly mediated by suppressing this pro‐atrophic signaling axis. This aligns with the known roles of MDPs in cytoprotection but highlights a specific efficacy in the context of glucocorticoid stress.

In addition, contrary to prevailing models, the atrophic response in our human myotubes occurred without detectable alterations in the apoptotic or inflammatory response. For instance, in rodent models DEXA has been shown to robustly increase caspase‐3 and NF‐κB activation (Chen et al., 2020; Lee et al., 2023), while these markers were unchanged in our human myotubes. these results highlight potential species‐specific differences and questions direct extrapolation from rodent models for predicting human muscular responses to glucocorticoids, thus arguing for the primacy of proteolytic pathway activation in the human context.

Against this human‐specific atrophic response to the glucocorticoid DEXA, MOTS‐c emerged as a powerful agent with potent anabolic and anti‐atrophic effects. On its own, MOTS‐c significantly increased both myotube area and fusion index, demonstrating a direct pro‐myogenic effect in agreement with previous work in both human immortalized myoblasts (LHCN‐M2) cell line and C2C12 cells (García‐Benlloch et al., 2022). Critically, when co‐administered with DEXA, MOTS‐c fully rescued the atrophic phenotype. This comprehensive rescue appears to be mediated by selective interference with the atrophic gene program, as MOTS‐c co‐treatment significantly attenuated the DEXA‐induced increase in MURF1 mRNA levels. Surprisingly, MOTS‐c did not mitigate the increase in MAFbx, suggesting that these two E3 ligases may be regulated by distinct upstream pathways and that MOTS‐c selectively targets the pathway leading to MURF1.

The observation that MOTS‐c alone enhanced myotube morphology by increasing area fraction and fusion index without affecting the mRNA levels of the investigated molecular markers suggests a dual, independent mechanism for this peptide. When co‐administered with dexamethasone, MOTS‐c acts primarily as an anti‐catabolic agent by suppressing the glucocorticoid‐induced upregulation of the E3 ligase, MURF1. However, the anabolic effect seen when MOTS‐c is used alone likely stems from higher Akt phosphorylation. This difference highlights that MOTS‐c's full therapeutic utility might rely on both its ability to block degradation and independently promote muscle building.

In parallel, this work provides the first evidence for HNG protective action against DEXA‐induced muscle atrophy. Although HNG alone did not modify myotube morphology, its co‐administration with DEXA rescued the myotube area fraction. This rescue was partial, as it did not extend to the fusion index, implying that HNG and MOTS‐c likely operate through distinct signaling pathways with HNG acting primarily to facilitate the preservation of pre‐existing myotube size rather than to enhance new myoblast fusion under these conditions.

We also observed that these effects were independent of various other major signaling pathways. For instance, both the atrophic actions of DEXA and the protective roles of the peptides were independent of myostatin signaling, as its expression did not change in any group. While DEXA can modify myostatin, the response is complex and dose‐ and time‐sensitive (Fappi et al., 2019; Wang et al., 2016). The inability of DEXA to modulate myostatin mRNA levels suggests that the increases in MURF1 and MAFbx expression at this time‐point occurred independently of myostatin signaling or that there is a delay in the regulation between those factors. Furthermore, the protective effect of MOTS‐c appears to be primarily through its interference with the ubiquitin proteasome system, without directly involving myostatin. The lack of effect of HNG and MOTS‐c on pathways such as NF‐κB and caspase‐3 is best interpreted as context‐dependent.

## 5 LIMITATIONS

We used in this study three experimental replicates (*n* = 3) across six treatment groups. Although typical in in vitro cell culture experiments, this small sample size limited our statistical power, particularly for variables with smaller effect sizes or greater inherent variability. A post‐hoc power analysis confirmed adequate power for variables with large effect sizes, such as myotube area fraction (88.8% power) and PGC1α expression (83.5% power). However, other variables, including fusion index (41.1% power), MAFbx expression (36.8% power), and most mitochondrial complex measurements (<20% power), were underpowered. Non‐significant findings for underpowered variables (e.g., myostatin levels, mitochondrial complexes) should be interpreted with caution due to a substantial risk of type II errors.

Despite those limitations, our findings offer valuable preliminary insights, particularly for variables with sufficient statistical power, providing a foundation for future confirmatory studies with larger sample sizes and more rigorous statistical methods.

## 6 CONCLUSION AND PERSPECTIVES

In conclusion, our investigation shows that in primary human myotubes, DEXA causes a strong atrophic phenotype marked by upregulation of MURF1 and MAFbx mRNA levels. However, the underpinning mechanisms differ radically from rodent models, in particular through a paradoxical upregulation of PGC‐1α and no alteration of canonical anabolic and inflammatory pathways. We find the mitochondrial‐derived peptide MOTS‐c to be a potent inhibitor that fully reverses this atrophy through selective inhibition of the upregulation of MURF1 by DEXA, and also by enhancing Akt signaling and suppressing STAT3 activation. HNG is partially protective, indicating separate mechanisms for these peptides. This study establishes MOTS‐c as a potent protective agent against glucocorticoid‐induced muscle atrophy in a relevant human cell model. Looking forward, future research should prioritize validating these findings in physiologically intact systems, such as appropriate in vivo models, to confirm efficacy and determine optimal systemic dosing. A critical next step involves elucidating the upstream, myostatin‐independent molecular mechanism through which MOTS‐c selectively targets the ubiquitin‐proteasome pathway, particularly its interaction with factors like KLF15 (Wang et al., 2017) Also, further investigations are warranted on the protein expression of atrophy‐related ligases such as MURF1. Successfully translating these MDPs means identifying their full therapeutic potential not only for glucocorticoid‐induced wasting but also for other severe muscle atrophy conditions, positioning MOTS‐c as an attractive and novel therapeutic candidate to mitigate sarcopenia and muscle loss associated with chronic disease.

## AUTHOR CONTRIBUTIONS

R.E., L.D., M.I., and S.R. conceived and designed research. R.E analyzed data, interpreted results of experiments, prepared figures and drafted manuscript. A.F., supported the statistical analysis and R.E., L.D., M.I., and S.R. edited and revised manuscript and approved final version of manuscript.

## FUNDING INFORMATION

The authors have nothing to report.

## CONFLICT OF INTEREST STATEMENT

The authors declare no conflicts of interest.

## ETHICS STATEMENT

The protocol was approved by the local Ethical Committee of the UCLouvain and conducted in accordance with the Declaration of Helsinki. The participants provided their written consent after being fully informed about the experimental procedure.

## Data Availability

Data will be made available upon reasonable request.
